# Fine scale relationships between sex, life history, and dispersal of masu salmon

**DOI:** 10.1002/ece3.228

**Published:** 2012-05

**Authors:** Shigeru Kitanishi, Toshiaki Yamamoto, Itsuro Koizumi, Jason B Dunham, Seigo Higashi

**Affiliations:** 1 College of Life Sciences, Ritsumeikan UniversityNoji-higashi, Kusatsu, Shiga 525-8577, Japan; 2 Department of Veterinary Nursing and Technology, Nippon Veterinary and Life Science UniversityMusashino, Tokyo 180-8602, Japan; 3 Creative Research Institution, Hokkaido UniversityN21W10 Sapporo, Hokkaido 001-0021, Japan; 4 U.S. Geological Survey, Forest and Rangeland Ecosystem Science CenterCorvallis, Oregon 97331; 5 Graduate School of Environmental Earth Science, Hokkaido UniversityN10W5 Sapporo, Hokkaido 060-0810, Japan

**Keywords:** Alternative strategy, gene flow, life-history polymorphism, microsatellite DNA, *Oncorhynchus masou*, sex-biased dispersal, spatial genetic structure

## Abstract

Identifying the patterns and processes driving dispersal is critical for understanding population structure and dynamics. In many organisms, sex-biased dispersal is related to the type of mating system. Considerably, less is known about the influence of life-history variability on dispersal. Here we investigated patterns of dispersal in masu salmon (*Oncorhynchus masou*) to evaluate influences of sex and life history on dispersal. As expected, assignment tests and isolation by distance analysis revealed that dispersal of marine-migratory masu salmon was male-biased. However, dispersal of resident and migratory males did not follow our expectation and marine-migratory individuals dispersed more than residents. This may be because direct competition between marine-migratory and resident males is weak or that the cost of dispersal is smaller for marine-migratory individuals. This study revealed that both sex and migratory life-history influence patterns of dispersal at a local scale in masu salmon.

## Introduction

Dispersal plays critical role in dynamics and structuring of populations (Dieckmann et al. 1999; Clobert et al. 2001). In theory, the propensity of individuals to disperse may depend on inbreeding avoidance (Pusey 1987) or competition for mates (Dobson 1982; Perrin and Mazalov 2000) or other resources (Greenwood 1980). The importance of these factors may differ between sexes, and consequently sex-biased dispersal is observed in many animals (Perrin and Mazalov 2000; Clobert et al. 2001). Hypotheses and predictions about sex-biased dispersal are dependent on the mating system of the species. In monogamous species, females tend to disperse more, because the benefits of philopatry are higher for males (Greenwood 1980; Perrin and Mazalov 2000; Fontanillas et al. 2004). Conversely, male-biased dispersal is expected in polygynous mating systems, because male reproductive success is limited by mating opportunities (Perrin and Mazalov 2000; Radespiel et al. 2003; Johansson et al. 2008).

Observations about sex-biased dispersal are consistent with hypotheses based on the species’ mating system, but there are exceptions (e.g., Kays et al. 2000; Consuegra and García de Leániz 2007), suggesting dispersal is also influenced by other factors. One such factor may be life-history polymorphism, which often involves different morphological features associated with dispersal ability (e.g., Clobert et al. 2001). Compared to the effects of sex, the effect of life-history variability on dispersal is poorly understood (e.g., Dawson et al. 2002). Here, we consider influences of both sex and migratory life history on dispersal of a salmonid fish. The mating system and migratory behavior of salmonid fishes provide an ideal setting for examining this issue.

Salmonid fishes are renowned for their extensive migrations and homing back to their natal streams to reproduce (Quinn and Dittman 1990). Whereas this general pattern is well-accepted, processes influencing local dispersal within natal streams are much less understood. The degree to which individuals disperse from their exact location of birth should depend on a variety of factors, including navigational error, habitat selection, and sexual selection (Dingle 1996). These influences should vary between sexes, as females must locate suitable spawning locations, and males must locate females with which to mate. Furthermore, in several species of salmon, males exhibit a variety of phenotypes associated with competition for access to females (Groot and Margolis 1991). In these species, some individual males forgo long-distance marine migrations to grow and mature within their natal stream. These freshwater resident individuals mature at sizes that are an order of magnitude smaller than their migratory cohorts and rely on sneak matings with females, since they stand little chance of success in competing directly against larger migratory males (Fleming and Gross 1994). In contrast to males, most females adopt a migratory life history, as body size is a critical factor influencing their fecundity and subsequent reproductive success (Jonsson and Jonsson 1993).

This scenario leads to several predictions about local dispersal in salmon. First, females should show stronger site fidelity and reduced dispersal than males, since natal homing has presumably evolved to direct females back to successful spawning locations (Cury 1994; Neville et al. 2006). Additionally, on the spawning grounds females almost exclusively participate in construction of spawning nests (redds; Groot and Margolis 1991; Esteve 2005). When spawning females occur at high local densities, they must defend redds from competitors. Redds left unguarded are vulnerable to superimposition by other females. These behaviors are believed to be particularly prevalent in semelparous species, where a female's lifetime reproductive success is often invested in a single redd. Overall, this suite of selective pressures on females should contribute to a reduced tendency of females to disperse, relative to males.

In the case of males, because individuals should seek as many mating opportunities as possible, intense competition for mates may lead to much higher levels of dispersal. It is more difficult, however, to predict whether dispersal should be greater for smaller “sneaker” males or larger and more dominant males. Presumably the success of either mating tactic is both density and frequency dependent (Rich et al. 2006), such that higher densities of either type should lead to greater dispersal (e.g., higher densities of dominant males should increase their propensity to disperse). The costs of movement may favor dispersal by larger migratory males, if dispersal by smaller resident males is limited by lower energy reserves and/or less capacity for swimming long distances (Kinnison et al. 2003). The long-distance migration and long life span of marine-migratory males may also increase possibility of dispersal relative to residents, due to increased likelihood of straying by migratory males seeking to return to their natal location. Accordingly, there are two processes that may favor greater dispersal by marine-migratory males: straying caused by failure to return to natal locations to reproduce, or localized movement or dispersal away from natal areas upon return. If the cost of dispersal is similar between resident and marine-migratory males, competition for mates should compel competitively inferior resident males to disperse to improve their fitness (Clobert et al. 2001). Although the roles of these factors are difficult to identify directly in the field, a recent study found that resident males tended to disperse more than marine-migratory males, and attributed the difference to competitive inferiority of the former (Olsen et al. 2006). However, the study did not fully separate the effects of sex and life history on dispersal (i.e., both sexes were pooled when the effects of life history were analyzed, and vice versa).

In this study, we sampled female and male masu salmon (*Oncorhynchus masou*) specifically to test predictions about hypothesized processes influencing local dispersal. We tested our predictions based on spatial patterns of genetic variability in males and females sampled in several locations within a single spawning stream network. We hypothesized that the importance of nest construction and guarding by females would reduce their dispersal relative to males overall. Within males, we predicted stronger philopatric tendency for larger migratory individuals, if smaller (and presumably competitively inferior) residents were forced to disperse more to find mates. Tests of these predictions were based on genetic assignments of individuals from different locations within the sampled stream network. We also evaluated general isolation by distance (IBD) among populations to test parallel predictions about local IBD between sexes and smaller versus larger males, with stronger IBD associated with more limited dispersal in females and marine-migratory males.

## Materials and Methods

### Study area and species

**Figure 1:**
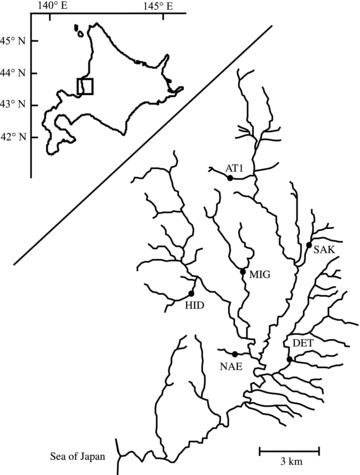
Sampling locations within six tributaries of the Atsuta River, Hokkaido, Japan.

The Atsuta River, located in Hokkaido, northernmost Japan (Fig. 1; 43°23′N, 141°25′E), is 136.5 km^2^ in watershed area and approximately 34 km long. This river has been closed to fishing for approximately 40 years, and there is no record of artificial release of masu salmon (Sugiwaka and Kojima 1984). In addition, this river has no artificial migration barriers such as weirs or dams, which are common in other rivers on Hokkaido. Therefore, relative to many streams in the region samples analyzed in this study were less influenced by anthropogenic effects and represented naturally reproducing wild fish.

Masu salmon inhabiting the Atsuta River are partially migratory, with marine-migratory and freshwater resident individuals present. In this river, some males (65–70% of males) exhibit marine migration and others (30–35% of males) remain in their natal river (i.e., freshwater resident form), whereas all females migrate to sea (Sugiwaka and Kojima 1984). Masu salmon exhibit a promiscuous mating system where single females simultaneously mate with multiple males, leading to intense male–male competition during spawning (Kato 1991; Kiso 1995). The lifespan of masu salmon varies between one to four years in this river and hence generations are overlapping (Sugiwaka and Kojima 1984). Like other salmonids, masu salmon show strong natal homing (Kato 1991), leading to the development of distinct populations within local spawning areas (Kitanishi et al. 2009). These conditions are ideal for the application of indirect methods for identification of dispersers (Paetkau et al. 2004).

### Sample collection

In 2003–2006, a total of 383 masu salmon individuals (marine-migratory males: 133; marine-migratory females: 130; resident males: 120) were collected from six tributaries of the Atsuta River, using electrofishing (Fig. 1; Table 1). Most of the marine-migratory individuals used in this study (250 of 263 individuals) were analyzed in Kitanishi et al. (2009). In this study, localized dispersal within tributaries was not analyzed independently because fidelity of individuals to specific natal locations could not be determined. In other words, patterns of dispersal revealed herein could represent the consequence of straying, local dispersal, or both processes acting simultaneously. Marine-migratory and resident individuals were easily distinguished based on the nonoverlapping body size distributions (Kato 1991). Marine-migratory males and females were also readily distinguished by secondary sexual characters. For DNA analyses, an adipose fin of each individual was removed and preserved in 99% ethanol at 3°C until DNA extraction.

**Table 1:** Sampling information and summary of genetic diversity indices of each sample of masu salmon sampled in the Atsuta River. Statistics reported are as follows: Ar refers to rarified (sample size corrected) within-population genetic diversity (El Mousadik and Petit 1996); *H*_E_ is expected heterozygosity; *F*_IS_ is the inbreeding coefficient of individuals, relative to the subpopulation or location (Weir and Cockerham 1984).

		*N*							
Sample	Sampling year	Distance from river mouth (km)	Anadromous male	Anadromous female	Resident male	Total	Ar^1^	*H*_E_^1^	*F*_IS_
AT1	2003–2005	28.9	22	24	18	64	9.8 [4.9, 14.8]	0.71 [0.56, 0.86]	0.033
SAK	2004–2006	22.2	17	21	21	59	9.5 [4.5, 14.4]	0.69 [0.53, 0.85]	0.003
HID	2003–2006	21.0	22	18	21	61	9.5 [4.4, 14.7]	0.69 [0.54, 0.83]	0.059
MIG	2004–2006	20.7	19	20	18	57	9.3 [4.7, 13.9]	0.69 [0.55, 0.84]	0.046
NAE	2005–2006	15.1	21	25	24	70	10.1 [4.5, 15.7]	0.71 [0.55, 0.86]	0.028
DET	2004–2006	13.0	32	22	18	72	10.1 [4.9, 15.0]	0.70 [0.55, 0.86]	−0.007

1 The 95% confidence intervals are given in brackets.

### Molecular analysis

Genomic DNA was extracted using Chelex 100 (Bio-Rad, Hercules, CA) according to the manufacturer's instructions. Nine microsatellite loci developed from other salmonids (One108, One110, One111, Olsen et al. 2000; OMM1325, OMM1344, OMM1347, Palti et al. 2002; OMM1148, Rexroad et al. 2002; OMM1375, OMM1402, Rodriguez et al. 2003) were amplified in an automated thermal cycler (polymerase chain reaction [PCR] thermal cycler SP, TAKARA, Otsu, Japan) in 10 μL of reaction mixture containing 0.5 unit of Taq polymerase, 1.5–2.5 mM MgCl_2_, 0.4–0.8 µM of each primer, 0.2 mM dNTP, 50 mM KCl, 20 mM Tris-HCl (pH 8.4), and 0.3–1.0 μL of genomic DNA as a template. For the PCR, thermal conditions of each primer were in accord with authors of each primer (see above). Subsequently, the PCR product was analyzed using ABI PRISM 3100 Genetic Analyser (Applied Biosystems, Foster City, CA), and fragment size data were collected using GeneScan Analysis version 3.7 (Applied Biosystems) followed by manual corrections.

Observed (*H*_O_) and expected (*H*_E_) heterozygosity were calculated for samples from each stream using GENEPOP 3.4 (Raymond and Rousset 1995). Within-population genetic diversity was calculated by the allelic richness corrected by the sample size (Ar; El Mousadik and Petit 1996), using FSTAT 2.9.3 (Goudet 2001). To examine the deviation from Hardy–Weinberg equilibrium (HWE) and linkage disequilibrium, we used exact HWE tests and composite linkage disequilibrium tests, as implemented in GENEPOP and FSTAT, respectively. Significance levels of the HWE and linkage disequilibrium tests were assessed following the false discovery rate (FDR; Benjamini and Yekutieli 2001). The extent of deviation from HWE was quantified by *F*_IS_ (Weir and Cockerham 1984) using FSTAT.

For analysis of population genetic structure, the genetic homogeneity and the extent of genetic differentiation among tributary samples were evaluated by Fisher's exact test and *F*_ST_, respectively. The significance levels of these tests were obtained by 10,000 permutations using GENEPOP and Arlequin 2.0 (Schneider et al. 2000), respectively, and were corrected following the FDR. IBD was also examined by regressing pairwise genetic distance on waterway distance (km). Pairwise stream distances were measured in each tributary and *F*_ST_/(1 –*F*_ST_) was used as the genetic distance (Rousset 1997). Correlations between genetic and geographic distance were evaluated using Mantel tests (Mantel 1967) with 10,000 permutations, using the program “ISOLDE” from GENEPOP.

### Biased dispersal

To assess differences in dispersal patterns between sexes and between life histories, indirect estimates were performed with three approaches, which are commonly used for sex-biased dispersal. To examine whether global tendency of dispersal patterns were different between sexes and between life histories, we first used the randomization method of Goudet et al. (2002), as implemented in the program “Biased Dispersal” from FSTAT. We calculated mean corrected assignment index (mAIc), variance of corrected assignment index (vAIc), *F*_ST_, and *F*_IS_ for groups of individuals, including marine-migratory males and females, and for marine-migratory males and resident males. The assignment index (AI) refers to the probability that an individual occurs within a sampling locality (Paetkau et al. 1995; Favre et al. 1997). The AIc is corrected by subtracting population means after log-transformation to remove population effect that may arise from different levels of genetic diversity in each population, and therefore the distribution of the AIc will be centered on zero (Favre et al. 1997). A positive value of AIc indicates an individual is philopatric and a negative value indicates a potential disperser (Goudet et al. 2002). Expected patterns of assignment and genetic differentiation paralleled Olsen et al. (2006). Because groups of individuals (i.e., males or females, and marine-migratory males or resident males) that have greater dispersal tendency would include both dispersers and residents, we interpreted groups with lower mAIc values to represent those with greater overall dispersal. Similarly, vAIc for the dispersing groups should be larger (Goudet et al. 2002). We interpreted groups with lower *F*_ST_ to represent those with greater overall dispersal. Finally, the sample with greater dispersal tendency should show a positive *F*_IS_ value due to the Wahlund effect. We interpreted increased *F*_IS_ to be indicative of increased dispersal within each group of individuals analyzed (Goudet et al. 2002; reviewed in Prugnolle and De Meeus 2002). In this procedure, differences in dispersal between sexes and life histories should be reflected in statistically significant dissimilarity in the estimated indices. We performed two-tailed tests by 10,000 permutations of the data to test the null hypothesis that dispersal of each form is equal.

Second, to evaluate the effect of sex and life history on dispersal, genetic dissimilarity and extent of genetic differentiation between samples that were grouped by sex and life history (i.e., marine-migratory males, marine-migratory females, and resident males) were quantified by Fisher's exact test and *F*_ST_, respectively. Statistical significance of these estimators was obtained by 10,000 permutations using GENEPOP and Arlequin. Significance of these tests was adjusted according to the FDR. Additionally, IBD of each sex and of each life history were examined by regressing pairwise genetic distance on stream distance (see section Molecular analysis). Furthermore, for comparison between sexes, we also investigated IBD of all males in which marine-migratory males and resident males were pooled.

Third, to infer whether each individual originated from the tributary in which it was sampled or was an immigrant from some other tributaries (i.e., disperser), we used a Bayesian estimator of dispersal and estimated the number of dispersers presented in the current generation. In this analysis, we conducted “Detection of first generation migrate” program implemented in GENECLASS 2 (Piry et al. 2004). In this approach, the likelihood for a given individual's genotype in a particular population was estimated. A disperser is defined as an individual that was not born into the tributary from which it was sampled, and this approach assigns each putative disperser to the most likely source population at a specific confidence level (Paetkau et al. 2004). The ratio *L*_home_:*L*_max_ was used as the statistical criterion to compute the likelihood of disperser detection (*L*), where *L*_home_ is the likelihood of drawing that individual's genotype from the population in which it was sampled, and *L*_max_ is the highest likelihood value among all population samples including the home population (i.e., the likelihood for the population to which the individual would be assigned). We used a Bayesian statistical approach (Rannala and Mountain 1997) with the Monte Carlo resampling method of Paetkau et al. (2004) to identify individuals as dispersers (Cornuet et al. 1999; Paetkau et al. 2004). The analysis was conducted with a simulation of 10,000 independent individuals (α= 0.05).

## Results

### Microsatellite variability

The exact test of HWE showed a significant global trend toward heterozygote deficiency (*P* < 0.001). Two samples showed significant deviation following FDR (*P*= 0.020, *k* [number of pairwise tests] = 6) and three out of 54 cases deviated significantly after FDR (*P*= 0.011, *k*= 54). This trend toward heterozygote deficiency was not caused by one particular locus, because Kendall's test of concordance showed that the ranks of all loci, as estimated by *F*_IS_, were not consistent across populations (Kendall's *W*= 0.229, *P* < 0.05). Linkage disequilibrium test suggested no significant linkages after FDR (*P*= 0.012, *k*= 36). Therefore, all loci were treated as independent and selectively neutral.

**Figure 2:**
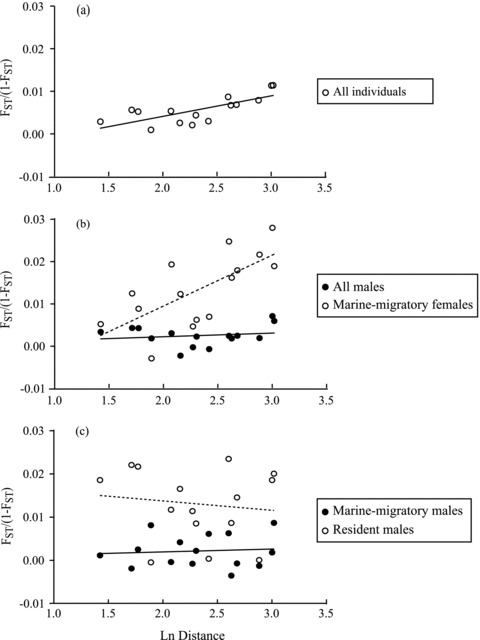
Slopes of IBD for six samples of masu salmon. (a) Includes plots and IBD slope of all individuals, regardless of sex or life history. (b) Separate plots and slopes of marine-migratory females (broken line) and all males (solid line). (c) Plots and slopes of marine-migratory males (solid line) and resident males (broken line). The relationship between genetic and geographic distance was statistically significant for all individuals and marine-migratory females.

Nine microsatellite loci were moderate to highly polymorphic in each population, with the number of alleles ranging from two (OMM1148) to 26 (One110). Allelic richness (Ar) within population ranged from 9.3 to 10.1 (Table 1). Mean expected heterozygosity (*H*_E_) was similar for each population, varying from 0.69 to 0.71 (Table 1). For pairwise comparisons, exact tests revealed 12 of 15 comparisons were significantly different at 5% level after FDR (Table S1). Although significant genetic differentiation (*F*_ST_) was also observed, with global *F*_ST_ of 0.011 (*P* < 0.001), the degree of genetic differentiation was low (ranged from 0.0009 to 0.0115, Table S1). The test for IBD revealed significant correlation between genetic and geographic distance (Fig. 2(a); all individuals, Mantel test: *P*= 0.012, *r*= 0.734, *y*= 0.0048*x*– 0.0055).

### Biased dispersal

In the analysis of global tendency of sex-biased dispersal, the four statistics were estimated separately for each sex (marine-migratory males vs. marine-migratory females, Table 2). Except for *F*_IS_, the trends in three other indices (mAIc, vAIc, *F*_ST_) were indicative of male-biased dispersal (i.e., lower mAIc, higher vAIc, lower *F*_ST_). Results from permutation tests rejected the null hypothesis that males and females disperse equally for both mAIc and *F*_ST_. In comparisons between life histories of males (marine-migratory males vs. resident males), all indices indicated that marine-migratory males had a higher tendency to disperse than resident males (i.e., lower mAIc, higher vAIc, lower *F*_ST_, and higher *F*_IS_, Table 2). The statistical test for mAIc and *F*_ST_ showed significant evidence for greater dispersal in marine-migratory individuals.

**Table 2:** Estimates of mAIc, vAIc, *F*_ST_, and *F*_IS_ for masu salmon samples from the Atsuta River by sex and life history. Probability values are from two-tailed tests.

Samples	*n*	mAIc	vAIc	*F*_ST_	*F*_IS_
Sex					
Marine-migratory males	133	−0.334	8.873	0.001	0.031
Marine-migratory females	130	0.342	7.263	0.012	0.046
*P*-value		0.027	0.161	0.007	0.720
Life history					
Marine-migratory males	133	−0.394	8.512	0.001	0.031
Resident males	120	0.437	8.469	0.014	−0.014
*P*-value		0.027	0.977	0.001	0.063

Significant genetic differentiation as estimated by *F*_ST_ and exact tests was observed only in marine-migratory females and resident males (Table S1). In marine-migratory males there was no significant differentiation (Table S1). We detected a clear pattern of IBD in marine-migratory females, suggesting significant negative correlation between dispersal and geographic distance (Fig. 2(b); *P*= 0.028, *r*= 0.686, *y*= 0.012*x*– 0.0146). In contrast, IBD was not significant when marine-migratory males and resident males were pooled (Fig. 2(b); all males, *P*= 0.393, *r*= 0.174, *y*= 0.0009*x*+ 0.0005). We also failed to detect relationships when marine-migratory males and resident males were treated separately (Fig. 2(c); marine-migratory males: *P*= 0.322, *r*= 0.09, *y*= 0.0007*x*+ 0.0006; resident males: *P*= 0.627, *r*=−0.129 *y*=–0.0022*x*+ 0.0181).

Using the Bayesian dispersal estimator, we found that most individuals were assigned to the tributary from which they were sampled. Twenty-nine out of 383 individuals (7.6% of all individuals) were identified as putative dispersers and the number of dispersers in each tributary ranged from two to seven (Table 3). The pattern of dispersal differed between sexes, with females more likely to be assigned to the tributary from which they were sampled. Among males, marine-migratory individuals were less likely to be assigned to their location of collection. In marine-migratory males, the number of dispersers was 14 (10.5%), whereas the number of dispersers in marine-migratory females and resident males were 8 (6.2%) and 7 (5.8%), respectively (Table 3).

**Table 3:** Number of dispersing masu salmon identified using a Bayesian genetic assignment test. Abbreviations correspond to sampled tributaries (first column) and its putative source populations (rows). Parenthetical values represent numbers of dispersers of marine-migratory male, marine-migratory female, and resident male, reading from left to right.

	Disperser	AT1	SAK	HID	MIG	NAE	DET
AT1	2	–		(1, 0, 0)	(0, 0, 1)		
SAK	7	(1, 0, 0)	–	(0, 1, 0)	(0, 0, 1)	(2, 0, 0)	(0, 0, 2)
HID	3			–	(0, 1, 0)	(0, 0, 1)	(0, 0, 1)
MIG	6	(2, 0, 0)	(1, 0, 0)		–	(2, 0, 0)	(0, 1, 0)
NAE	7	(2, 0, 0)	(1, 1, 0)	(0, 1, 0)	(0, 1, 0)	–	(0, 1, 0)
DET	4	(1, 1, 0)			(1, 0, 0)	(0, 0, 1)	–

## Discussion

We found male masu salmon dispersed more than migratory females. This agrees with the prediction that male-biased dispersal is favored in a promiscuous mating system (Perrin and Mazalov 2000). In a promiscuous mating system, the reproductive success of males is limited by mating opportunity, and hence males would compete with other males for females. Promiscuous mating, lack of paternal care (Kato 1991), and strong male-biased operational sex ratio of masu salmon (Koseki and Maekawa 2000; Yamamoto and Edo 2006) would reinforce competition among males, and individuals should seek as many mating opportunities as possible. Thus, dispersal would be favored among males, especially for competitively inferior individuals.

However, although some studies have found male-biased dispersal (Hard and Heard 1999; Hutchings and Gerber 2002; Bekkevold et al. 2004; Neville et al. 2006), others have failed to detect it (Unwin and Quinn 1993; Olsen et al. 2006; Consuegra and García de Leániz 2007). Two nonexclusive explanations may exist. First, species- and/or population-specific life-history polymorphisms may affect dispersal pattern. In salmonids, life-history traits such as homing, occurrence of partial migration, age at reproduction, and sex ratio can vary dramatically in space or time (e.g., Kato 1991; Hendry and Stearns 2004). A second consideration is the difference in geographic scale (extent) investigated in each study. Most of the studies that have documented sex-biased dispersal have been conducted at greater extents, involving comparisons among major rivers (Hard and Heard 1999; Bekkevold et al. 2004; Fraser et al. 2004), rather than local dispersal within more limited extents (Neville et al. 2006). Given the differing selective pressures presumably acting on males versus females, as well as between migratory and resident males, we would expect the results of studies of dispersal could be scale-dependent. The effect of scale combined with the importance of local conditions such as age structure (Hard and Heard 1999) or density (Palstra et al. 2007; Gomez-Uchida et al. 2009) should be expected to produce wide variability among studies examining dispersal. We conclude that it is difficult to compare results of different studies of local dispersal in salmonids because the results of each may be so strongly influenced by variation in these multiple influences.

Another finding of this study is that dispersal was linked to migratory life history: marine-migratory males tended to disperse more than resident males. Based on the hypothesis that dispersal is associated with presumed competitive inferiority of small resident males (Clobert et al. 2001), we initially expected that resident-biased dispersal might occur in masu salmon. Olsen et al. (2006) tested this prediction in a comparison of two populations of marine-migratory and freshwater resident rainbow trout and found it to be partially supported. Comparisons between our results and Olsen et al. (2006) are complicated by potential behavioral differences between the species, as well as the ecological context. For example, unlike masu salmon, small resident females are present in rainbow trout, which may be forced to disperse by marine-migratory females for competing limited spawning sites (Olsen et al. 2006 pooled both sexes when assessing the effect of life-history forms). Another possibility is that a male-biased operational sex ratio of masu salmon in the Atsuta River may be strong, and resulting in greater competition among marine-migratory males could have driven more individuals to disperse, relative to resident males. Furthermore, in contrast to rainbow trout (e.g., McMillan et al. 2007), marine-migratory male masu salmon appear to be indifferent to resident males during mating (Koseki and Maekawa 2000; Yamamoto and Edo 2002). This suggests that direct competition between life histories may not influence dispersal of smaller resident males.

Alternatively, the difference in dispersal between marine-migratory males and resident males could simply be tied to greater ability of larger marine-migratory individuals to move. If this is true of masu salmon, the limited ability of smaller individuals to move may constrain their dispersal (Kinnison et al. 2003), thus, explaining patterns we observed here. A tendency toward greater dispersal in marine-migratory males may be reinforced by the fact that some resident males are iteroparous, while all marine-migratory males are semelparous (Kato 1991; Kiso 1995). In the case of marine-migratory males, it would be critical to obtain successful matings because they will die after the spawning season. Accordingly, individuals may be more likely to disperse to seek successful matings.

Finally, it is possible the homing ability of marine-migratory masu salmon males may be reduced relative to freshwater resident males. Marine-migratory males move over far greater distances within their lifetimes, and spawn at a later age relative to freshwater males (i.e., greater length of time; Sugiwaka and Kojima 1984). These two factors should increase the probability that marine-migratory males would exhibit reduced ability to faithfully return to their natal spawning locations (i.e., increased probability of navigation error or straying). In contrast, homing may not even be necessary for freshwater males if they remain in or near these locations within their lifetimes.

This study revealed that both sex and migratory life history influence patterns of dispersal as revealed by population genetic structuring at a local scale in masu salmon. Our prediction of greater site fidelity in females was confirmed. This, combined with lack of IBD in males indicates that genetic differentiation in the Atsuta River can be attributed to females. Conversely, males may be agents of dispersal and possibly gene flow. Few dispersal studies have considered the effects of life-history differences, which our results suggest could be considerable. Accordingly, sampling and analysis of samples for fine-scale population structuring should be mindful of these potential influences and account for them whenever possible. Combining individuals with different dispersal characteristics could lead to increased variance within samples caused by unintentional pooling of individuals with different spatial patterns of divergence. In addition, sampling of only one life history could lead a misleading impression of the overall genetic structure. Our study was limited in terms of sample sizes and numbers of loci considered (see Goudet et al. 2002), but we were still able to detect influences of individual characteristics on population structuring.

## Supporting Information - Evolutionary Analysis

**Table S1.** Pairwise *F*_ST_ (below diagonal) and the significance of allelic homogeneity (above diagonal) of each type of masu salmon among six samples in the Atsuta River.
